# Intimate partner violence identified through routine antenatal screening and maternal and perinatal health outcomes

**DOI:** 10.1186/s12884-019-2527-9

**Published:** 2019-10-16

**Authors:** Karina Chaves, John Eastwood, Felix A. Ogbo, Alexandra Hendry, Bin Jalaludin, Sarah Khanlari, Andrew Page

**Affiliations:** 1 Albury Wodonga Health, Albury, New South Wales Australia; 2 Department of Community Paediatrics, Sydney Local Health District, Croydon Community Health Centre, Croydon, New South Wales Australia; 3 School of Women’s and Children’s Health, The University of New South Wales, Kensington, Sydney, New South Wales Australia; 4 Ingham Institute for Applied Medical Research, UNSW, Liverpool New South Wales, Australia; 5 Community Health Services, Sydney Local Health District, Level 9, King George V Building, Missenden Road, Camperdown, NSW 2050 Australia; 6 Sydney Institute for Women, Children and their Families, Camperdown, NSW 2050 Australia; 7 Charles Perkins Centre, Menzies Centre for Health Policy, Discipline of Child and Adolescent Health, and School of Public Health, University of Sydney, Camperdown, NSW 2006 Australia; 8 Translational Health Research Institute, School of Medicine, Campbelltown Campus, Western Sydney University, Penrith, NSW Australia; 9 Coverage and Surveillance, National Centre for Immunisation Research and Surveillance, The Children’s Hospital at Westmead, Locked Bag 4001, Westmead, NSW 2145 Australia; 10 Healthy People and Places Unit, South Western Sydney Local Health, Liverpool NSW, Australia; 11 Department of Community Child Health/Integrated Care, Sydney Children’s Hospital Network, Randwick, New South Wales Australia

**Keywords:** Intimate partner violence, Prenatal screening, Antenatal, Postnatal depression, Breastfeeding, Australia

## Abstract

**Background:**

This study investigated the association between intimate partner violence (IPV) identified on routine prenatal screening and perinatal outcomes for mother and infant.

**Methods:**

Routinely collected perinatal data for a cohort of all women and their infants born in public health facilities in Sydney (Australia) over the period 2014–2016 (*N* = 52,509) were analysed to investigate the risk of adverse maternal and perinatal outcomes associated with a history of IPV. The association between an affirmative response on prenatal IPV screening and low birth weight (LBW) < 2.5 kg, preterm birth < 37 weeks, breastfeeding indicators and postnatal depressive symptoms (PND) was investigated in a series of logistic regression models.

**Results:**

IPV was associated with an increased risk of PND (OR = 2.53, 95% CI 1.76–3.63), not breastfeeding at birth (OR = 1.65, 95% CI 1.30–2.09), non-exclusive breastfeeding at discharge (OR = 1.66, 95% CI 1.33–2.07) and first post-natal visit (OR = 1.54, 95% CI 1.24–1.91). Self-reported fear of a partner was strongly associated with an increased risk of PND (OR = 3.53, 95% CI 2.50–5.00), and also LBW (OR = 1.58, 95% CI 1.12–2.22), preterm birth (OR = 1.38, 95% CI 1.08–1.76), lack of early initiation of breastfeeding (OR = 1.67, 95% CI 1.28–2.17), non-exclusive breastfeeding at discharge from hospital (OR = 1.60, 95% CI 1.24–2.06) and at the first post-natal visit (OR = 1.27, 95% CI 0.99–3.04).

**Conclusions:**

IPV reported at the time of pregnancy was associated with adverse infant and maternal health outcomes. Although women may be disinclined to report IPV during pregnancy, universal, routine antenatal assessment for IPV is essential for early identification and appropriate management to improve maternal and newborn health.

## Introduction

Intimate-partner violence (IPV) is defined as physical violence, sexual violence, stalking or psychological aggression by a current or former partner. IPV affects 1 to 28% of pregnant women worldwide, with the majority of studies indicating a prevalence of 3–9%, [1] with a higher reported prevalence among the general population of women (~ 30%), and a higher proportion of IPV among lower (compared to higher) income regions [2].

IPV screening, routinely conducted in the Australian context, [3] has been shown to increase the identification rates of women experiencing IPV, although this does not result in a commensurate increase in referrals to specialist services, with the long-term health benefits to women unknown [4]. Two recent meta-analyses have found that women who experience IPV during pregnancy are at increased risk of preterm birth and low birth weight neonates [5, 6]. Heterogeneous study findings could be attributed to differences in the assessment of IPV exposure and the selection of study populations, with subsequent limitations to generalisability of the findings. These previous studies have reported an association between IPV during pregnancy and increased risk of preterm birth and low birth weight, but with limitations relating to selection and recall bias.

Previous studies examining the impact of IPV among non-pregnant women have been conducted in the Australian context, and have focussed on post-traumatic psychiatric morbidity [7] and sustained physical injuries [8, 9]. The few Australian studies that have reported on IPV at the time of pregnancy, indicate a weak or no association between IPV during pregnancy and adverse birth outcomes [10–12].

In New South Wales (NSW), Australia, mandatory routine IPV screening in prenatal clinics commenced in 2001 with the aim of increasing identification of IPV, ensuring early specialist referral and the promotion of strategic interventions. Accordingly, this study aims to investigate the association between recent or current IPV and the maternal and perinatal outcomes of postnatal depressive symptoms, low birth weight, preterm birth and exclusive breastfeeding.

## Methods

### Data source

This study used a retrospective perinatal data set of all live births for the period 2014–2016 (*N* = 52,509) in public health facilities in Sydney Local Health District (SLHD) and South Western Sydney Local Health District (SWSLHD) (a total of 7 hospitals). Maternal prenatal data including socio-demographic information, history of previous pregnancies, history of maternal tobacco or alcohol use and history of IPV were collected at the first prenatal care visit by qualified midwives across both health districts. Postnatal data were collected at the post-birth visit by qualified nurses and were stored in the Information Management & Technology Division (IM&TD) database of each health district. Both prenatal and postnatal data were obtained with ethics approval and were linked, using unique individual identifiers. Ethics approvals were obtained from the South Western Sydney Local Health District and Sydney Local Health District Ethics Committees to conduct data linkage provided that data remained anonymous. (Approval numbers HREC: LNR/11/LPOOL/463; SSA: LNRSSA/11/LPOOL/464 & Project No: 11/276 LNR; Protocol No X17–0454 & LNR/12/RPAH/266).

### Study setting

The geographic area of SWSLHD and SLHD captures approximately 52% of the Sydney metropolitan region and represents a population catchment of approximately 1,621,000 people. The study population captures approximately 80% of all women who had live births in the geographic area during the study time period, excluding those who opted to have their obstetric care delivered in the private sector. SWSLHD and SLHD are ethnically diverse, with more than a third of people born outside Australia, and represents heterogeneous socio-economic characteristics.

### Intimate partner violence

The main exposure variables were (i) self-reported physical abuse by an intimate partner in the previous 12 months and (ii) self-reported fear of a partner or ex-partner at the time of pregnancy. This information is routinely collected at the first obstetric care visit for women according to the NSW Routine Domestic Violence Screening Policy. Women are asked:

(i) “Within the last year have you been hit, slapped or hurt in other ways by your partner or ex-partner?”
(ii) “Are you frightened of your partner or ex-partner?”

These two questions were taken from the Abuse Assessment Screen (AAS), a brief validated screening tool that is used extensively among pregnant women [13]. The NSW Routine Domestic Violence Screening questions have been shown to increase identification of women experiencing IPV in prenatal clinics in Australia and evaluation has shown the questions are deemed acceptable by most women [3].

IPV was categorized as a binary variable. An affirmative answer for question (i) was considered to be an indicator of recent IPV. An affirmative answer to question (ii) was considered to be an indicator of current fear of a partner or ex-partner. Women were not screened, as per NSW Health protocol, if another person, including the partner, was present, if the woman declined, if the woman was not well enough to answer questions, or where an interpreter was not available (for women whose first language was not English) (total missing responses was 8848 (16.9%) and 9165 (17.5%) for questions (i) and (ii) respectively).

### Outcome variables

A series of outcome variables were defined relating to birth outcomes, postnatal depressive symptoms and exclusive breastfeeding. Each outcome was selected for their demonstrated impact on infant and childhood physical and neurocognitive development. The outcome data were collected according to the New South Wales Perinatal Data Collection guidelines, which is a population-based surveillance system covering all births in NSW public and private hospitals, as well as home births [14].

### Birth outcomes

Low birth weight (LBW) was defined as singleton birth weight < 2.5 kg with the binary outcome being normal singleton birth weight ≥ 2.5 kg. Premature birth was defined as a live birth < 37 weeks gestation with the binary outcome being term birth ≥37 weeks. Gestational age at birth was specified in the electronic neonatal record and based on an estimation from the first day of the last menstrual period, maternal dating ultrasound or clinical examination of the infant as per NSW Perinatal Collection guidelines.

### Breastfeeding indicators

Breastfeeding outcomes were treated as binary variables and were derived at three-time points – initiation of breastfeeding at the time of birth, exclusive breastfeeding at the time of discharge from hospital and exclusive breastfeeding at the first postnatal visit (between 0 and 6 weeks, with the first postnatal visit occurring in 0–2 weeks among approximately 66% of women). Early initiation of breastfeeding was defined as the introduction of breastmilk within the first hour of birth. Exclusive breastfeeding was defined as infants aged 0–5 months who received only breastmilk (including expressed breastmilk) and included infants who required oral rehydration solution or medications. Infant feeding definitions were based on National Health and Medical Research Council (NHMRC) infant feeding guidelines, [15] consistent with the World Health Organization definitions for assessing infant feeding practices [16]. Non-exclusive breastfeeding was defined as infants aged 0–5 months who received other liquids such as infant formula, water or water-based juices.

### Postnatal depression

Postnatal depressive symptoms were based on the Edinburgh Postnatal Depression Scale (EPDS) [17] which was completed at the first postnatal visit within the first six weeks after birth. The EPDS rates the severity of depressive symptoms in the preceding seven days exploring items such as dysphoric mood, anxiety, guilt and suicidal thoughts. The number of depressive symptoms was calculated to obtain a total score out of 30. EPDS scores were coded as a categorical variable with scores > 12 indicating significant depressive symptomatology [18]. The EPDS is a validated screening tool within Australia, [19] and can be applied in multiple languages [20].

### Statistical analyses

The association between previous IPV and self-reported fear of partner or ex-partner was investigated in a series of logistic regression models for the following outcomes: (i) low birth weight (< 2.5 kg) (ii) preterm birth (< 37 weeks) (iii) lack of initiation of breastfeeding at birth, lack of exclusive breastfeeding at discharge from hospital and at the first postnatal visit and (iv) postnatal depressive symptoms (EPDS > 12). Univariate models investigated the association between positive screening for physical IPV or self-reported fear of partner and perinatal outcomes, followed by multivariate models incorporating the following confounders: antenatal depressive symptoms, maternal age, socio-economic status, indigenous status, maternal body mass index (BMI), alcohol consumption during pregnancy, smoking during pregnancy, history of antenatal medical problems and intervention at birth.

### Missing data

**Fig. 1:**
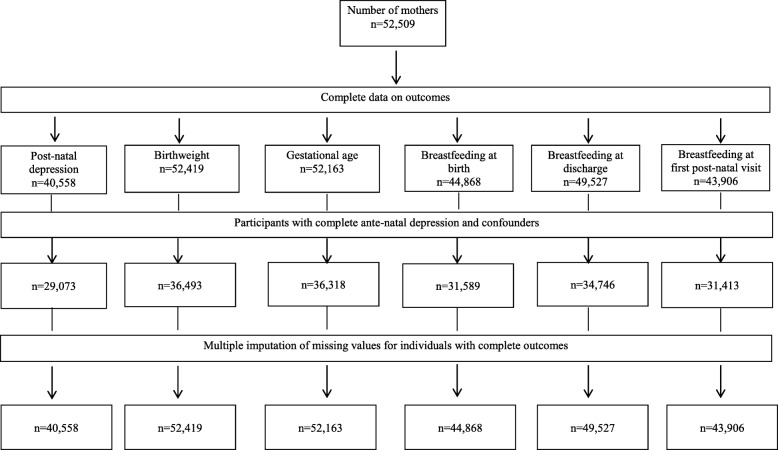
Flow chart of available data on self-reported domestic violence, birth outcomes, and breast feeding behaviours in Central and South Western Sydney mothers (*N* = 17,564) of new born children (2014)

Missing data analyses using multivariate imputation by chained equations (MICE) [21] were conducted to investigate the potential impact of incomplete information on study factors and confounders in a series of sensitivity analyses (Fig. 1). MICE assumes that known characteristics of participants can be used to estimate the characteristics of individuals with missing data and that data are Missing At Random (MAR) [22]. Revised measures of association are estimated from imputed data, which can then be compared with associations observed in the complete case analysis. The imputed dataset was based on the original cohort comprising complete outcome data for each outcome of interest. All outcome and study variables in the analysis as described above were included in the multiple imputation modelling, as well as additional variables available on the dataset. These additional variables included BMI, Apgar score, language spoken at home, hospital of birth, maternal history of child abuse, offspring sex, baby birth weight in grams and gestational age in weeks. Multiple imputation was conducted using the *ice* command in Stata (Stata Corp, V.14.0, College Station, TX, USA) and based on 20 multiple imputations [23]. Revised odds ratios were generated using the *mim* command to combine estimates across the 20 multiply imputed datasets.

## Results

The prevalence of self-reported IPV at the time of pregnancy was 1.8% (95% CI: 1.7–1.9%) and self-reported fear of partner was 1.4% (95% CI: 1.3–1.5%) in the cohort. The prevalence of LBW was 5.9% and premature birth was 13.8%. The prevalence of PND was 3.1% (Table 1). The prevalence of delayed initiation of breastfeeding at birth was 10.1%, while the prevalence of non-exclusive breastfeeding at discharge from hospital was 10.5 and 16.2% at the first postnatal visit (Table 2).

**Table 1:** Prevalence of self-reported domestic violence and birth outcomes by study factor

	Post-natal depression			Low birth weight			Gestational age		
	Cases	Participants	%	Cases	Participants	%	Case	Participants	%
Total	1272	40,558	3.1	3075	52,419	5.9	7213	52,163	13.8
Physical abuse by partner									
No	826	28,596	2.9	1627	35,865	4.5	4311	35,696	12.1
Yes	33	477	6.9	41	628	6.5	84	622	13.5
Frightened of partner									
No	822	28,679	2.9	1631	35,996	4.5	4314	35,823	12.0
Yes	37	394	9.4	37	497	7.4	81	495	16.4
Ante-natal depressive symptom									
No	594	27,361	2.2	1524	34,381	4.4	4096	34,213	12.0
Yes	265	1712	15.5	144	2112	6.8	299	2105	14.2
Maternal age-group									
< 20 years	787	27,128	2.9	1516	34,018	4.5	4013	33,859	11.9
20–39 years	60	1497	4.0	112	1911	5.9	299	1901	15.7
> 40 years	12	448	2.7	40	564	7.1	83	558	14.9
SES category									
High	394	11,411	3.5	713	14,770	4.8	1765	14,692	12.0
Middle	380	14,370	2.6	794	17,478	4.5	2130	17,395	12.2
Low	85	3292	2.6	161	4245	3.8	500	4231	11.8
Australian-born									
No	342	13,734	2.5	748	17,014	4.4	2042	16,913	12.1
Yes	517	15,339	3.4	920	19,479	4.7	2353	19,405	12.1
BMI category									
Underweight	458	15,965	2.9	938	20,021	4.7	2291	19,949	11.5
Normal Weight	50	1711	2.9	168	2176	7.7	300	2160	13.9
Overweight	220	6687	3.3	329	8357	3.9	995	8306	12.0
Obese	131	4710	2.8	233	5939	3.9	809	5903	13.7
Ante-natal problems									
No	704	24,699	2.9	1300	30,801	4.2	3510	30,646	11.5
Yes	155	4374	3.5	368	5692	6.5	885	5672	15.6
Intervention at birth									
No	722	25,471	2.8	1122	31,943	3.5	3408	31,788	10.7
Yes	137	3602	3.8	546	4550	12.0	987	4530	21.8
Alcohol consumption									
No	850	28,622	3.0	1648	35,919	4.6	4325	35,745	12.1
Yes	9	451	2.0	20	574	3.5	70	573	12.2
Smoking									
No	486	27,018	1.8	1431	33,679	4.2	3962	33,521	11.8
Yes	73	2055	3.6	237	2814	8.4	433	2797	15.5

**Table 2:** Prevalence of self-reported domestic violence and breast feeding indicators by study factor

	No breastfeeding at birth			No breastfeeding at discharge			No breastfeeding at first postnatal visit		
	Cases	Participants	%	Cases	Participants	%	Cases	Participants	%
Total	4516	44,868	10.1	5213	49,527	10.5	1464	9053	16.2
Domestic violence in previous 12 months									
No	826	28,596	2.9	1627	35,865	4.5	4311	35,696	12.1
Yes	33	477	6.9	41	628	6.5	84	622	13.5
Domestic violence: scared of partner									
No	822	28,679	2.9	1631	35,996	4.5	4314	35,823	12.0
Yes	37	394	9.4	37	497	7.4	81	495	16.4
Ante-natal depressive symptom									
No	594	27,361	2.2	1524	34,381	4.4	4096	34,213	12.0
Yes	265	1712	15.5	144	2112	6.8	299	2105	14.2
Maternal age-group									
< 20 years	787	27,128	2.9	1516	34,018	4.5	4013	33,859	11.9
20–39 years	60	1497	4.0	112	1911	5.9	299	1901	15.7
> 40 years	12	448	2.7	40	564	7.1	83	558	14.9
SES category									
High	394	11,411	3.5	713	14,770	4.8	1765	14,692	12.0
Middle	380	14,370	2.6	794	17,478	4.5	2130	17,395	12.2
Low	85	3292	2.6	161	4245	3.8	500	4231	11.8
Australian-born									
No	342	13,734	2.5	748	17,014	4.4	2042	16,913	12.1
Yes	517	15,339	3.4	920	19,479	4.7	2353	19,405	12.1
BMI category									
Underweight	458	15,965	2.9	938	20,021	4.7	2291	19,949	11.5
Normal Weight	50	1711	2.9	168	2176	7.7	300	2160	13.9
Overweight	220	6687	3.3	329	8357	3.9	995	8306	12.0
Obese	131	4710	2.8	233	5939	3.9	809	5903	13.7
Ante-natal problems									
No	704	24,699	2.9	1300	30,801	4.2	3510	30,646	11.5
Yes	155	4374	3.5	368	5692	6.5	885	5672	15.6
Intervention at birth									
No	722	25,471	2.8	1122	31,943	3.5	3408	31,788	10.7
Yes	137	3602	3.8	546	4550	12.0	987	4530	21.8
Alcohol intake code									
No	850	28,622	3.0	1648	35,919	4.6	4325	35,745	12.1
Yes	9	451	2.0	20	574	3.5	70	573	12.2
s									
No	486	27,018	1.8	1431	33,679	4.2	3962	33,521	11.8
Yes	73	2055	3.6	237	2814	8.4	433	2797	15.5

IPV was associated with not breastfeeding at birth (OR = 1.65, 95% CI: 1.30–2.09, *P* = < 0.001), non-exclusive breastfeeding at discharge (OR = 1.66, 95% CI: 1.33–2.07, *P* < 0.001) and at the first post-natal visit (OR = 1.54, 95% CI: 1.24–1.91, *P* < 0.001), following adjustment for confounders in the complete case analysis (Table 3). IPV was associated with postnatal depressive symptoms (OR = 2.53, 95% CI: 1.76–3.63, *P* < 0.001), and to a lesser extent LBW (OR = 1.34, 95% CI: 0.97–1.85, *P* = 0.077) and premature birth (OR = 1.09, 95% CI: 0.86–1.38, *P* = 0.464) (Table 3). Analyses of imputed data for missing confounders showed a similar pattern of associations, with slightly stronger associations evident between physical IPV and study outcomes.

**Table 3:** Associations between self-reported experience of physical abuse in the past 12-months and post-natal depression, birth outcomes, and breast feeding behaviours in South Western Sydney and Sydney Local Health Districts in 2014 (*N* = 17,564)

				Complete case			Multiple imputation.				
	n	N	%	OR (95% CI) (a)	*P* value	OR (95% CI) (b)	*P* value	OR (95% CI) (a)	*P* value	OR (95% CI) (b)	*P* value
Postnatal depressive symptom											
Experience of domestic violence											
No	826	28,596	2.9	1.00		1.00		1.00		1.00	
Yes	33	477	6.9	2.50 (1.74–3.58)	< 0.001	2.53 (1.76–3.63)	< 0.001	3.11 (2.28–4.24)	< 0.001	3.11 (2.28–4.24)	< 0.001
Low birth weight (< 2500 g)											
Experience of domestic violence.											
No	1627	35,865	4.5	1.00		1.00		1.00		1.00	
Yes	41	628	6.5	1.47 (1.07–2.03)	0.019	1.34 (0.97–1.85)	0.077	1.70 (1.32–2.20)	< 0.001	1.57 (1.21–2.02)	0.001
Gestational age (< 37 weeks)											
Experience of domestic violence.											
No	4311	35,696	12.1	1.00		1.00		1.00		1.00	
Yes	84	622	13.5	1.14 (0.90–1.43)	0.279	1.09 (0.86–1.38)	0.464	1.32 (1.06–1.64)	0.014	1.26 (1.02–1.57)	0.035
No early initiation of breast feeding											
Experience of domestic violence											
No	3168	31,076	10.2	1.00		1.00		1.00		1.00	
Yes	88	513	17.2	1.82 (1.45–2.30)	< 0.001	1.65 (1.30–2.09)	< 0.001	1.90 (1.52–2.37)	< 0.001	1.65 (1.31–2.07)	< 0.001
Non-EBF at discharge.											
Experience of domestic violence.											
No	3617	34,183	10.6	1.00		1.00		1.00		1.00	
Yes	103	563	18.3	1.89 (1.52–2.35)	< 0.001	1.66 (1.33–2.07)	< 0.001	2.01 (1.64–2.47)	< 0.001	1.71 (1.39–2.12)	< 0.001
Non-EBF at first post-natal visit											
Experience of domestic violence											
No	4633	30,895	15.0	1.00		1.00		1.00		1.00	
Yes	122	518	23.6	1.75 (1.42–2.14)	< 0.001	1.54 (1.24–1.91)	< 0.001	1.93 (1.60–2.33)	< 0.001	1.66 (1.36–2.02)	< 0.001

Notes: (a): Unadjusted Odds Ratio (b): Adjusted for maternal age, socio-economic status, Indigenous status, alcohol consumption during pregnancy, smoking during pregnancy, body mass index, history of antenatal problems, antenatal depression, and intervention during birth

The magnitude of associations between self-reported fear of intimate partner at the time of pregnancy were slightly stronger for study outcomes, compared to physical intimate partner violence (Table 4). Self-reported current fear of partner was strongly associated with postnatal depressive symptoms (OR = 3.53, 95% CI: 2.50–5.00, *P* < 0.001) following adjustment for confounders (Table 4). Similarly, self-reported fear of partner was associated with not initiating breastfeeding at birth (OR = 1.67, 95% CI 1.28–2.17, *P* < 0.001), non-exclusive breastfeeding at discharge from hospital (OR = 1.60, 95% CI: 1.24–2.06, *P* < 0.001) and at the first postnatal visit (OR = 1.27, 95% CI: 0.99–1.64, *P* = 0.060), following adjustment for confounders (Table 4). Self-reported fear of partner was associated with premature birth (OR = 1.38, 95% CI: 1.08–1.76, *P* = 0.009), and LBW (OR = 1.58, 95% CI: 1.12–2.22, *P* = 0.009) (Table 4). Analyses of imputed data for missing confounders showed a similar pattern of associations between self-reported fear of partner and study outcomes.

**Table 4:** Associations between self-reported fear of partner or ex-partner and post-natal depression, birth outcomes, and breast feeding behaviours in South Western Sydney and Sydney Local Health Districts in 2014 (N = 17,564)

				Complete case			Multiple imputation.				
	n	N	%	OR (95% CI) (a)	*P* value	OR (95% CI) (b)	*P* value	OR (95% CI) (a)	*P* value	OR (95% CI) (b)	*P* value
Postnatal depressive symptom											
Fear of partner or ex-partner											
No	822	28,679	2.9	1.00		1.00		1.00		1.00	
Yes	37	394	9.4	3.51 (2.49–4.96)	< 0.001	3.53 (2.50–5.00)	< 0.001	4.06 (2.94–5.59)	< 0.001	4.04 (2.93–5.58)	< 0.001
Low birth weight (< 2500 g)											
Fear of partner or ex-partner											
No	1631	35,996	4.5	1.00		1.00		1.00		1.00	
Yes	37	497	7.4	1.69 (1.21–2.38)	0.002	1.58 (1.12–2.22)	0.009	1.79 (1.36–2.35)	< 0.001	1.65 (1.25–2.17)	< 0.001
Gestational age (< 37 weeks)											
Fear of partner or ex-partner											
No	4314	35,823	12.0	1.00		1.00		1.00		1.00	
Yes	81	495	16.4	1.43 (1.12–1.82)	0.004	1.38 (1.08–1.76)	0.009	1.52 (1.21–1.91)	< 0.001	1.45 (1.15–1.82)	0.002
No early initiation of breast feeding											
Fear of partner or ex-partner											
No	3185	31,166	10.2	1.00		1.00		1.00		1.00	
Yes	71	423	16.8	1.77 (1.37–2.29)	< 0.001	1.67 (1.28–2.17)	< 0.001	1.83 (1.46–2.30)	< 0.001	1.63 (1.29–2.06)	< 0.001
Non-EBF at discharge.											
Fear of partner or ex-partner											
No	3642	34,288	10.6	1.00		1.00		1.00		1.00	
Yes	78	458	17.0	1.73 (1.35–2.21)	< 0.001	1.60 (1.24–2.06)	< 0.001	1.92 (1.53–2.40)	< 0.001	1.69 (1.33–2.13)	< 0.001
Non-EBF at first post-natal visit.											
Fear of partner or ex-partner											
No	4672	30,996	15.1	1.00		1.00		1.00		1.00	
Yes	83	417	19.9	1.40 (1.10–1.78)	0.006	1.27 (0.99–1.64)	0.060	1.68 (1.36–2.08)	< 0.001	1.45 (1.16–1.81)	< 0.001

Notes: (a): Unadjusted Odds Ratio (b): Adjusted for maternal age, socio-economic status, Indigenous status, alcohol consumption during pregnancy, smoking during pregnancy, body mass index, history of antenatal problems, antenatal depression, and intervention during birth

## Discussion

This study showed an association between intimate partner violence at the time of pregnancy and an increased risk of LBW, premature birth, postnatal depressive symptoms and delayed initiation of breastfeeding and non-exclusive breastfeeding.

The prevalence of physical IPV experienced within the past year by women and reported at the time of pregnancy in this study was 1.8% and self-reported current fear of partner was 1.4% which is lower than the prevalence of IPV in pregnancy recorded in previous studies in the United States [24–26] and Australia [10, 11, 27]. The variation in prevalence estimates can be attributed to different measures to assess IPV exposure and differences in the ethnic and socioeconomic characteristics of the population samples. Variation in IPV prevalence likely reflects recall bias associated with self-reported measures of IPV, different reference periods for reporting IPV (e.g. period versus lifetime prevalence measures), and which may also be differential by socio-demographic groups and population samples. Janssen et al., [28] conducted a population-based study in Canada (*N* = 4750) which used similar IPV screening questions to the current study and found comparable prevalence for physical IPV and self-reported fear of partner, and found a similar association for pre-term delivery.

Findings linking IPV at the time of pregnancy and an increased risk of LBW and premature birth are also consistent with recent meta-analyses [5, 6] which showed an association between IPV during pregnancy and LBW and premature birth. One of the meta-analyses explored associations between physical and sexual IPV [5] unlike our study which did not explore sexual IPV specifically.

Many of the previous studies that showed an association between IPV and study outcomes were cross-sectional or case-control designs. Previous cohort studies that showed an association also differed from the present study, in that they were conducted in lower-income populations, [29] recruited smaller population samples [30]. or selected population samples presenting to hospital or police after reporting IPV [10, 31, 32]. Other cohort studies have not shown an association between IPV at the time of pregnancy and low birth weight and preterm birth [33]. The present study is one of the largest population-based cohort studies to find an association between IPV and birth outcomes, despite its low prevalence in the cohort. This prevalence is likely an under-estimate of IPV in this population, and likely reflects under-reporting perhaps due to a fear that the partner may find out that IPV has been reported, or that health or community services may intervene.

Our finding of an association between IPV reported at the time of pregnancy and postnatal depressive symptoms was consistent with previous studies. Unlike previous studies, [34–36] this strong association did not attenuate substantially following adjustment for confounders (including antenatal depressive symptoms). Antenatal depressive symptoms has also been shown in this population sample to be associated with adverse birth outcomes [37].

The findings that physical IPV and self-reported fear are associated with failure to initiate and sustain exclusive breastfeeding has also been previously reported [38]. This finding is in contrast to previous Australian [39] and American [40] studies which have investigated the association between IPV in pregnancy and breastfeeding outcomes, but did not show a consistent association in analyses when adjusting for confounders (ORs ~ 0.7 to 1.5). The present study found that self-reported fear of partner at the time of pregnancy had a stronger association with adverse outcomes compared to self-reported physical abuse in the previous 12 months. Current self-reported fear of partner could be perceived by women to relate to a broader range of experience of IPV which includes emotional, physical and/or sexual abuse. Combined types of IPV in pregnancy have been shown to have stronger associations with adverse perinatal outcomes [5, 6]. Additionally, the exposure of current fear of partner in pregnancy was more proximate to birth outcomes, compared to the screening question of previous physical abuse, which may have increased the associations with study outcomes.

The findings of the current study have important public health implications given that IPV is estimated to affect a third of women worldwide. Women who experience current self-reported fear of intimate partner in pregnancy are more likely to deliver an infant at biological risk for premature birth or LBW, potentially leading to poorer neurodevelopmental outcomes for children. Women who experience IPV in pregnancy are also more likely to experience PND. PND creates an environmental risk for adverse neurodevelopmental outcomes by impacting on infant attachment, feeding, behavioural regulation and long-term cognitive development [41, 42]. Breastfeeding can be a protective factor in establishing mother-infant attachment and other infant health outcomes [43] but this study shows that this is less likely to occur if IPV has been experienced perinatally.

In NSW, Australia, health professionals follow a specified protocol on how to respond to women disclosing IPV at the time of prenatal screening. Responses include risk assessment, safety planning, information and referral to domestic violence support services. Our findings would urge health professionals to ensure evidence-based interventions targeting antenatal depression, PND and breastfeeding difficulties are included in perinatal care services for women identified on prenatal IPV screening.

Evidence of the effectiveness of current interventions in reducing and preventing domestic violence for women, as well as impacts on maternal and infant health outcomes, has not been clearly demonstrated [44, 45]. Barriers to providing effective responses to those experiencing IPV are varied and may include psychiatric comorbidity, distrust of services and lack of multi-agency collaboration. Women experiencing IPV often access multiple agencies including legal, police, financial, housing, child protection, health and domestic violence services for themselves and their children. Models of care that incorporate integrated, multi-agency teams to provide ‘wrap-around’ services for vulnerable mothers and infants are promising in this setting and evaluation of these interventions for women experiencing IPV would be worthwhile.

This study has a number of strengths. Firstly, the cohort sample size was large and based on a community sample limiting the possibility of selection bias and allowing generalization to other Australian states and territories. Secondly, the potential selection bias due to missing data was investigated in a sensitivity analysis, which showed that missing data were unlikely to have affected the magnitude of associations substantially. Thirdly, some of our outcome measures were based on validated tools in our screening population (EPDS) or were objective measures of infant health outcomes (e.g. LBW, gestational age).

Our study has several limitations. Firstly, the method to assess IPV exposure was based on the validated Abuse Assessment Screen, however questions chosen for the NSW Routine Domestic Violence Screen have not been independently validated. Our study would support the recommendations by Spangaro et al. [45] that a validated screening tool for IPV in pregnancy be piloted in the Australian population and compared to the current screening tool. Secondly, methods to assess breastfeeding were based on a combination of direct observation by maternity unit staff and self-report. This could lead to reporter and measurement bias in quantifying exclusive breastfeeding and affect the magnitude of associations found.

Thirdly, it is likely that there are a number of unmeasured confounders that may have impacted on study outcomes such as multi-parity, substance abuse and experience of IPV in the postnatal period. Previous research has shown a high correlation between IPV experienced in the prenatal and postnatal periods [46]. Data on exposure to IPV by women are routinely collected during the postnatal period in our study population and would be useful in future analyses.

## Conclusion

Women who report IPV at the time of pregnancy are at increased risk for development of depressive symptoms and their infants are at biological and environmental risk for poorer developmental outcomes. Health professionals who identify IPV during prenatal screening should be mindful of the likely impact of postnatal depression and breastfeeding difficulties on the mother-child dyad. For women experiencing IPV in pregnancy, further research into multi-agency integrated care models that provide assistance and support throughout the perinatal period is needed.

## Data Availability

The data that support the findings of this study are available from South Western Sydney Local Health District and Sydney Local Health District but restrictions apply to the availability of these data, which were used under license for the current study, and so are not publicly available. Data are however available from the authors upon reasonable request and with permission of the NSW Ministry of Health Data Custodian.
